# Automatic algorithm for quantifying lung involvement in patients with chronic obstructive pulmonary disease, infection with SARS-CoV-2, paracoccidioidomycosis and no lung disease patients

**DOI:** 10.1371/journal.pone.0251783

**Published:** 2021-06-10

**Authors:** Allan Felipe Fattori Alves, José Ricardo Arruda Miranda, Fabiano Reis, Abner Alves Oliveira, Sérgio Augusto Santana Souza, Carlos Magno Castelo Branco Fortaleza, Suzana Erico Tanni, José Thiago Souza Castro, Diana Rodrigues Pina

**Affiliations:** 1 Botucatu Medical School, Clinics Hospital, Medical Physics and Radioprotection Nucleus, Botucatu, SP, Brazil; 2 Institute of Bioscience, Sao Paulo State University Julio de Mesquita Filho, Botucatu, SP, Brazil; 3 Radiology and Medical Imaging, State University of Campinas, Campinas, SP, Brazil; 4 Medical School, Sao Paulo State University Julio de Mesquita Filho, Botucatu, SP, Brazil; Northwestern University Feinberg School of Medicine, UNITED STATES

## Abstract

In this work, we aimed to develop an automatic algorithm for the quantification of total volume and lung impairments in four different diseases. The quantification was completely automatic based upon high resolution computed tomography exams. The algorithm was capable of measuring volume and differentiating pulmonary involvement including inflammatory process and fibrosis, emphysema, and ground-glass opacities. The algorithm classifies the percentage of each pulmonary involvement when compared to the entire lung volume. Our algorithm was applied to four different patients groups: no lung disease patients, patients diagnosed with SARS-CoV-2, patients with *chronic obstructive pulmonary disease*, and patients with *paracoccidioidomycosis*. The quantification results were compared with a semi-automatic algorithm previously validated. Results confirmed that the automatic approach has a good agreement with the semi-automatic. Bland-Altman (B&A) demonstrated a low dispersion when comparing total lung volume, and also when comparing each lung impairment individually. Linear regression adjustment achieved an R value of 0.81 when comparing total lung volume between both methods. Our approach provides a reliable quantification process for physicians, thus impairments measurements contributes to support prognostic decisions in important lung diseases including the infection of SARS-CoV-2.

## Introduction

Computed tomography has been used as a standard procedure in the diagnosis of many lung diseases: acute pulmonary embolism, chronic pulmonary hypertension, interstitial lung disease, lung infection, bronchial carcinoma, and emphysema [1]. More recently, CT imaging has been recommended as the main method for the diagnosis of SARS-CoV-2 [2], which can affect more than 50% of the lung. Knowing the impact of lung diseases worldwide, the search for more effective and less invasive methods to characterize the extent of lung involvement is essential [3–7].

In the last two decades, CT scanners increased their spatial and temporal resolution. Multi-detector scanners can acquire up to hundreds of 1-mm slices simultaneously [8]. It allowed the characterization and quantification of anatomic structures with more confidence [9]. The advantage of CT examination compared to other modalities is that it is a fast exam, it provides lung anatomy in sectional slices, allows the 3D volumetric reconstruction and it provides the early detection of abnormalities.

The identification of pulmonary structure is often done subjectively by radiologists. Changes in the pulmonary structures represent progress or not of obstructive and restrictive respiratory diseases. Therefore, its response corresponds to different therapeutic alternatives. Quantification measurements are difficult to obtain and relies on subjective estimations of lung volume and the size of the compromised area within the lung. Radiological evidence is generally classified based on the experience of each radiologist. Such analyzes are subject to intra and interobserver variations [10].

Image processing has been used to aid the diagnosis of radiologists. The precise and detailed quantification of lung volume through image processing is an important part of the construction of the diagnosis of lung diseases. In this context, the objective quantification of pulmonary structures through CT is an important and indispensable tool for diagnosis and therapeutic evaluation in clinical practice [11–17].

Quantification is important in the assessment of diseases, such as Chronic Obstructive Pulmonary Diseases (COPD), which lead to an increase in the residual lung volume [4, 18], paracoccidioidomycosis (PCM) [17], and SARS-CoV-2 [2], demonstrating abnormal patterns as an inflammatory process, fibrosis, emphysema, ground-glass, and others. And also, of great importance when a reduction in lung volume occurs due to diseases that cause pulmonary focal obstruction of a given airway.

In this work, we aimed to develop a practical tool for the quantification of total lung volume and lung impairments in different diseases. We developed an automatic algorithm for those quantifications using high resolution computed tomography (HRCT) exams. The algorithm was capable of measuring volume and differentiating pulmonary involvement including inflammatory process and fibrosis, emphysema, and ground-glass opacities. The algorithm classified the percentage of each pulmonary involvement when compared to the entire lung volume. Our algorithm was applied to four different patients groups: no lung disease patients, patients diagnosed with SARS-CoV-2, patients with COPD, and patients with PCM. The quantification results were compared with a semi-automatic algorithm previously validated [17].

## Methods

### Patient selection

The study was developed with ethical approval from São Paulo State University Ethics Comitee and CONEP—National Commission on Ethics in Research (Protocol Number 8773 / *Certificado de Apresentação de Apreciação Ética CAAE* 83901617.3.0000.5411). All data were fully anonymized before access thus informed consent was dismissed. All HRCT examinations were performed at the Diagnostic Imaging Service of Botucatu Medical School, Sao Paulo State University and Campinas Medical School, University of Campinas, Brazil.

The research involved a set of 172 retrospective HRCT exams pre-classified with defined diagnosis distributed among: no lung disease patients, patients with coronavirus disease (SARS-CoV-2 infection), chronic obstructive pulmonary disease (COPD), and paracoccidioidomycosis (PCM). The description of each group and the inclusion and exclusion criteria is presented below.

#### No lung disease patients

We included 62 HRCT scans of No lung disease patients (45 men and 25 women). The mean age of the patients was 52.2 ± 13.9, selected by an experienced radiologist with over 20 years of experience.

*Inclusion criteria*. No lung disease patients without significant changes in lung that could compromise normal lung volume from the radiological point of view. Adult individuals who underwent chest CT scans for the most diverse indications, in the suspicion of thoracic alterations or as a complement to the assessment of extrathoracic diseases.

*Exclusion criteria*. Patients with pulmonary parenchymal diseases, atelectasis, pulmonary hyperinflation or hypertransparence, lung masses, pleural diseases or mediastinal masses, deformities of the thoracic bone framework, any retractable or expansive process of the wall, pleural, pulmonary or mediastinal. These data were acquired in our archive of images (all of them obtained before December 2019).

#### SARS-CoV-2 infection patients

We included 31 retrospective HRCT scans for patients with SARS-CoV-2 (18 men and 13 women), without any previous pulmonary pathology. The mean age of the patients was 59.8 ±17.3 and there were no specific days of symptoms to perform CT exams.

*Inclusion criteria*. Adult patients with confirmation of SARS-CoV-2 through real-time Polymerase Chain Reaction (RT-PCR), 3 to 6 days after hospitalization.

*Exclusion criteria*. Patients with immunosuppression (transplantation, chemotherapy) and HIV-positive and previous diseases with lung involvement.

#### Patients with chronic obstructive pulmonary disease (COPD)

We included 37 patients with COPD (20 men and 17 women). The mean age of the patients was 68.7 ± 13.9.

*Inclusion criteria*. Patients with COPD who experienced desaturation on exertion during sleep, accompanied by physicians. The diagnosis of COPD was assessed utilizing a post-bronchodilator spirometry exam with a Forced Expiratory Volume in the first second (FEV1) / Forced Vital Capacity (FVC) <0.70, according to criteria of national and international guidelines [19].

*Exclusion criteria*. Patients with severe hypoxemia (PaO2≤55mmHg), polycythemia, cancer on any site, active smokers, and those who had an exacerbation in the last three months before the initial assessment were identified. These data were acquired in our archive of images (all of them obtained before December 2019).

#### Patients with paracoccidioidomycosis (PCM)

We included 42 patients with PCM (39 men and 3 women). The mean age of the patients was 49.8 ± 12.3.

*Inclusion criteria*. Patients with PCM confirmed by the identification of P. brasiliensis, and / or, only with the presence of serum anti-P. brasiliensis antibodies determined by the double immunodiffusion reaction on agar gel, which present paracoccidioid pulmonary involvement. We included patients who presented the chronic form of PCM with pulmonary involvement characterized by respiratory complaints and chest radiography with interstitial and / or alveolar lesions.

*Exclusion criteria*. Patients with the presence of any other disease that could compromise the lungs, other systemic diseases, and aggravating factors, except smoking and alcoholism. These data were acquired in our archive of images (all of them obtained before December 2019).

### Image acquisition

Chest CT exams without contrast were evaluated in the study. Images were acquired as retrospective HRCT scans on a multislice CT scanner Toshiba Activision 16 (Toshiba America Medical Systems, Tustin, CA) with the following parameters: the pixel size ranged from 0.59mm x 0.59 mm to 0.80 mm x 0.80 mm, 515 x 512 pixel matrix size, 5.0 mm increment between slices. 5.0 mm slice with a tube voltage of 120 kV. Axial sections (1-mm thickness) were obtained at 10-mm interval throughout the entire chest, with around 300 slices acquired for each patient. All analyzed images were acquired in the inspiration phase.

### Pulmonary quantification

All patients in our database passed through algorithms for segmentation and quantification of different regions in the lung. In this work, two different algorithms were utilized. The first algorithm uses a completely automatic approach for segmentation, which means that it does not require any steps commanded by users. The second algorithm uses a semi-automatic approach with a radiologist as an operator. The semi-automatic was validated in a previous study by Alvarez, de Pina et al. 2014 [17]. The main difference between the two algorithms relies on the segmentation steps. After segmentation, both algorithms follow the same scheme of quantification procedures better described in the following sections.

### Automatic algorithm

The automatic algorithm was developed using Matlab R2017a (Mathworks, Natick, MA, USA) for the quantification of pulmonary involvement on CT exams. For all data processing, we used a personal computer with a Quad-Core CPU with 3.4 GHz, 16 GB of RAM, Windows 10 operating system, and 2 TB Hard Drive. The algorithm follows a segmentation process based on thresholding and morphological operators. The main steps are described below.

**Fig 1:**
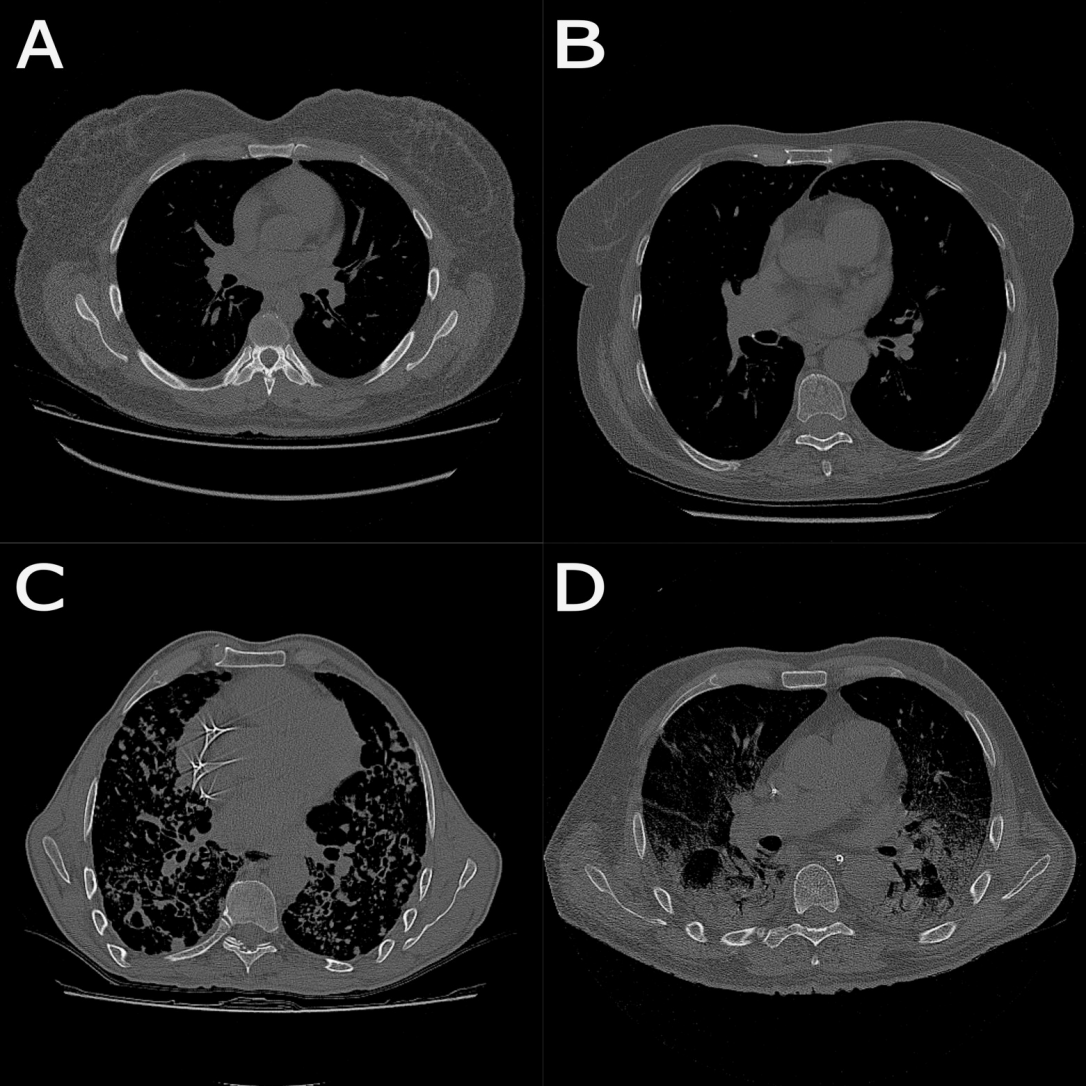
Different slices of CT scans representing each patients group. A–*No lung disease*, B–COPD, C–PCM and D—SARS-CoV-2.

**Fig 2:**
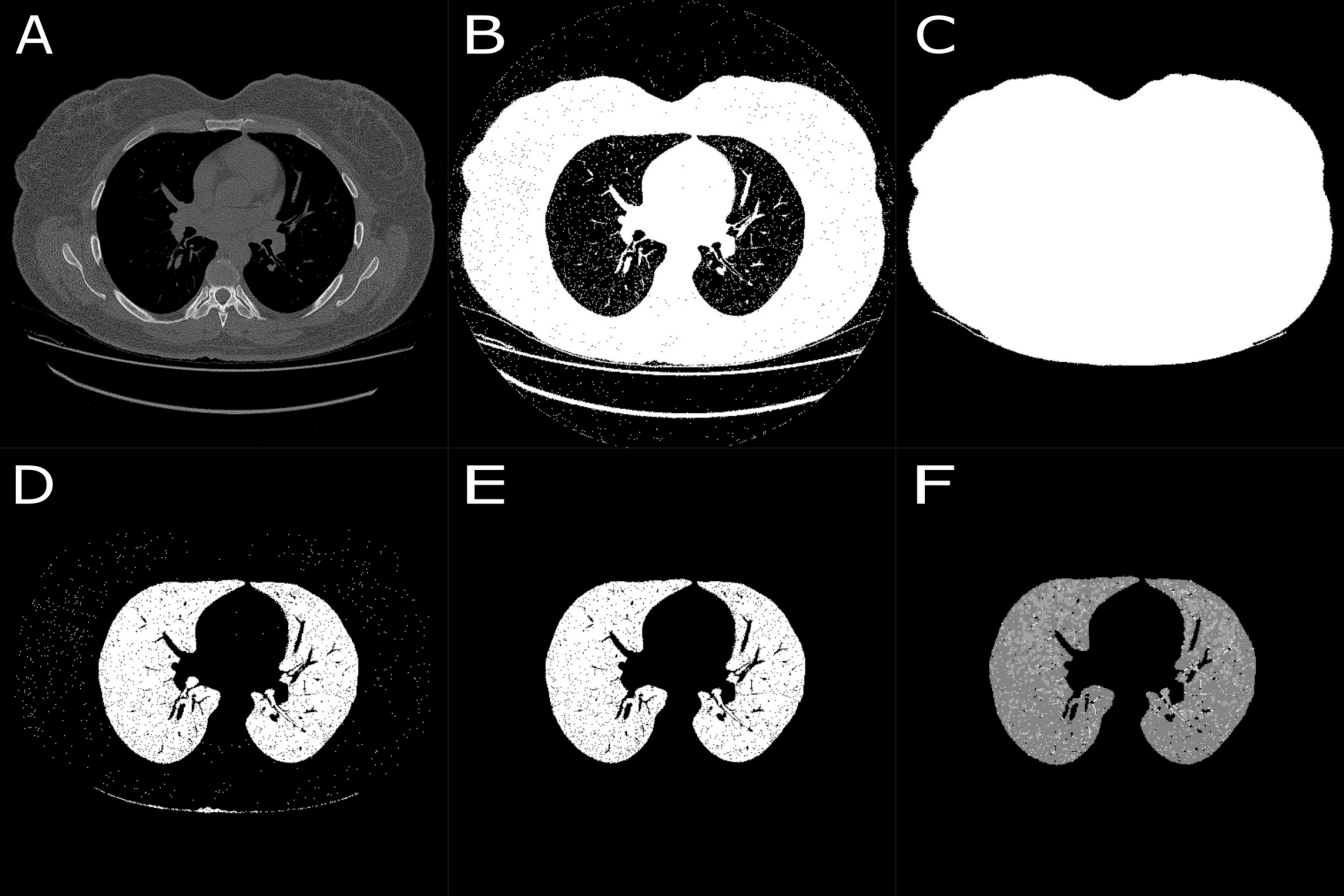
Sequence of images that demonstrate the algorithm steps. A–Original image; B–First thresholding; C–Body segmentation; D–Second Threshold; E–Lung Segmentation and F–Final segmentation.

1. All images in the CT scan sequence are read. Fig 1 exemplifies a single slice of each patient group included in the study, No lung disease, B- COPD, C- PCM and D- SARS-COV-2.
2. Fig 2 shows sequences performed by the algorithm in the middle of a patient’s lung. It can be seen in Fig 2A (as an example a slice of a No lung disease patient). A first threshold is performed to select the range of pixels including all tissues, as can be seen in Fig 2B. Then, the largest area is selected, which comprises the patient’s body, and thus air, bed, and other external structures are excluded, depicted in Fig 2C.
3. A second threshold is applied to highlight regions with Hounsfield units close related to lung tissues, as can be seen in Fig 2D.
4. In the sequence, two morphological operators and a filter are applied to the image (fill, median filter, and dilation) to confirm the selected regions filled with air, while softening surrounding tissues.
5. The two largest areas are selected, composed by the lungs, thus excluding the trachea and other adjacent soft tissues demonstrated in Fig 2E.
6. A second dilation performs an expansion of the pulmonary region, in cases where peripheral fibrosis occurs.
7. Then different thresholds are applied for each type of tissue of interest: emphysema, inflammatory process and fibrosis and ground-glass opacities, resulting in the image seen in Fig 2F.
8. For each tissue, its areas within each slice are estimated, based on the number and area of the pixels.
9. In the sequence, the same operations are applied to all slices that compose the image sequence of that patient.

Total lung volumes, emphysema volume, inflammatory process/fibrosis volume, and ground-glass volume are then computed. Regarding the application of both algorithms, the thresholds for quantification were as follows: emphysema: -1500 to -920 Hounsfield Units (HU); Ground-glass Opacities: -595 to-5 HU; and fibrosis and inflammatory process -5 to 450 HU. All volumes were measured in mm^3^. The inflammatory process and fibrosis were quantified as the same impairment. This occurs since both impairments average pixel values (Hounsfield Units) are similar [17].

**Fig 3:**
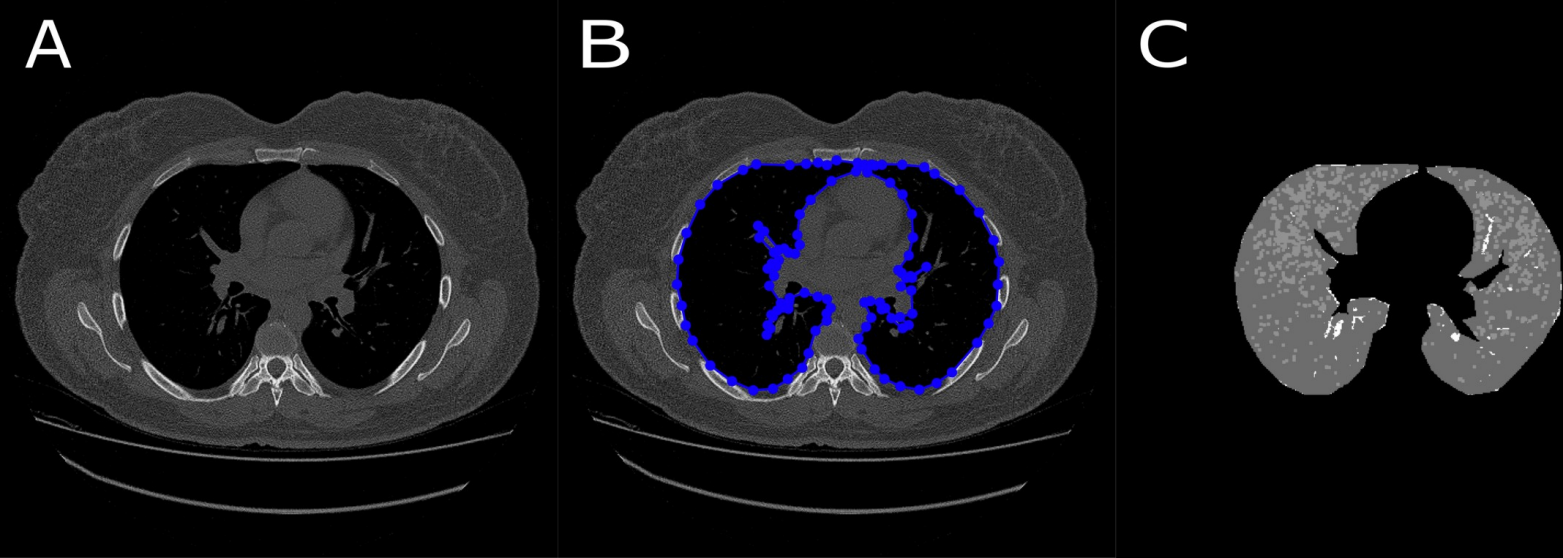
Sequence of images that demonstrate the semi-automatic algorithm steps. A–Original image; B–Segmentation performed by an experienced radiologist. All blue lines and dots represent the manual segmentation of lung boundaries. C–Final segmentation.

The same examinations were analyzed through the semi-automatic algorithm. The process is called semi-automatic since the segmentation steps are performed manually by an experienced radiologist [17]. Fig 3 exemplifies the application of the semi-automatic algorithm. In this step, the operator (experienced radiologist) manually determines the lung boundaries with the mouse cursor. All patients were analyzed by the same operator to prevent possible interobserver variability. All the data were measured in mm^3^, thus defining a volumetric region of interest. The operator performs the segmentation with a step of five slices. Similar to the automatic algorithm, total lung volumes, emphysema volume, inflammatory process/fibrosis volume, and ground-glass volume are then counted through the same thresholds.

The validation of this methodology was previously described by Alvarez, de Pina et al. 2014 [17]. In that paper, virtual phantoms were constructed for algorithm validation. Different amounts of emphysema and inflammatory process tissues were introduced on a normal lung tissue background. The involved regions were filled with a pseudorandom gray level determined by a Gaussian distribution. The error was determined from the difference between the exact value implemented in the phantom and computational value generated through the algorithm. In that validation process, the greatest error encountered was 3.4% among all virtual phantoms [17].

### Statistical analysis

Comparison between volumes of the total lung, inflammatory process and fibrosis, emphysema, and ground-glass opacities were performed using Bland-Altman statistics to assess agreement between the automatic and semi-automatic method. To demonstrate the agreement in volume between automatic and semi-automatic approaches we utilized linear regression and the Bland-Altman (B&A) method. B&A describes the agreement between two quantitative measurements, with well-established limits of agreement. Limits are estimated from the mean and standard deviation of the differences between the two quantities. In an XY scatter plot, the difference between the two quantities is plotted against the mean. A good result occurs when 95% of the data points lie within ± 2 standard deviations [20].

## Results

The selection of patients according to our criteria of inclusion and exclusion resulted in 172 HRCT scans. As described in the automatic algorithm steps, each stage contributes to the segmentation of the lung in each CT slice. Table 1 depicts the results for the 172 patients’ examinations with all quantification results from both algorithms. Each column demonstrates the estimations of the lung volume, percentage of inflammatory process and fibrosis in relation to the total volume of the patient, percentage of emphysema in relation to the total volume of the patient, and ground-glass opacities in relation to the total volume of the patient. Ground-glass opacities were not found in COPD and No lung disease patients. All other impairments are demonstrated with their mean value and standard deviation.

**Table 1:** Quantification values for semi-automatic (S-A) and automatic (A) algorithms, with mean and standard-deviation for lung volume, inflammatory process and fibrosis volume, emphysema volume, and ground-glass opacities volume.

Group	S-A Volume (10^5^ mm^3^)	A Volume (10^5^ mm^3^)	S-A Inflammatory Process and Fibrosis (%)	A Inflammatory Process and Fibrosis (%)	S-A Emphysema	A Emphysema	S-A Ground-glass	A Ground-glass
COPD	1.11 ±0.19	1.06 ±0.21	3.37± 0.55	5.64 ±3.26	44.4 ±21.54	49.38 ±22.59	0	0
No lung disease	1.01 ±0.18	0.93 ±0.2	3.54 ±0.92	6.52 ±3.87	11.44 ±9.36	13.57 ±10.39	0	0
SARS-CoV-2	1.86 ±0.31	1.81 ±0.49	12.5± 8.3	9.80± 2.9	11.73 ±10.48	10.4 ±8.48	41.44 ±16.77	43.74 ±11.77
PCM	1.87 ±0.24	2.34 ±0.46	4.70± 3.5	8.50 ±4.2	7.82 ±6.05	7.16 ±5.3	13.59 ±5.66	21.63 ±5.5
**Total Mean +/- SD**	**1.4625 ±0.4**	**1.53 ±0.54**	**6.03 ±3.24**	**7.62±1.54**	**18.85 ±12.78**	**20.12 ±14.62**	**27.52 ±13.93**	**32.69 ±11.06**

**Fig 4:**
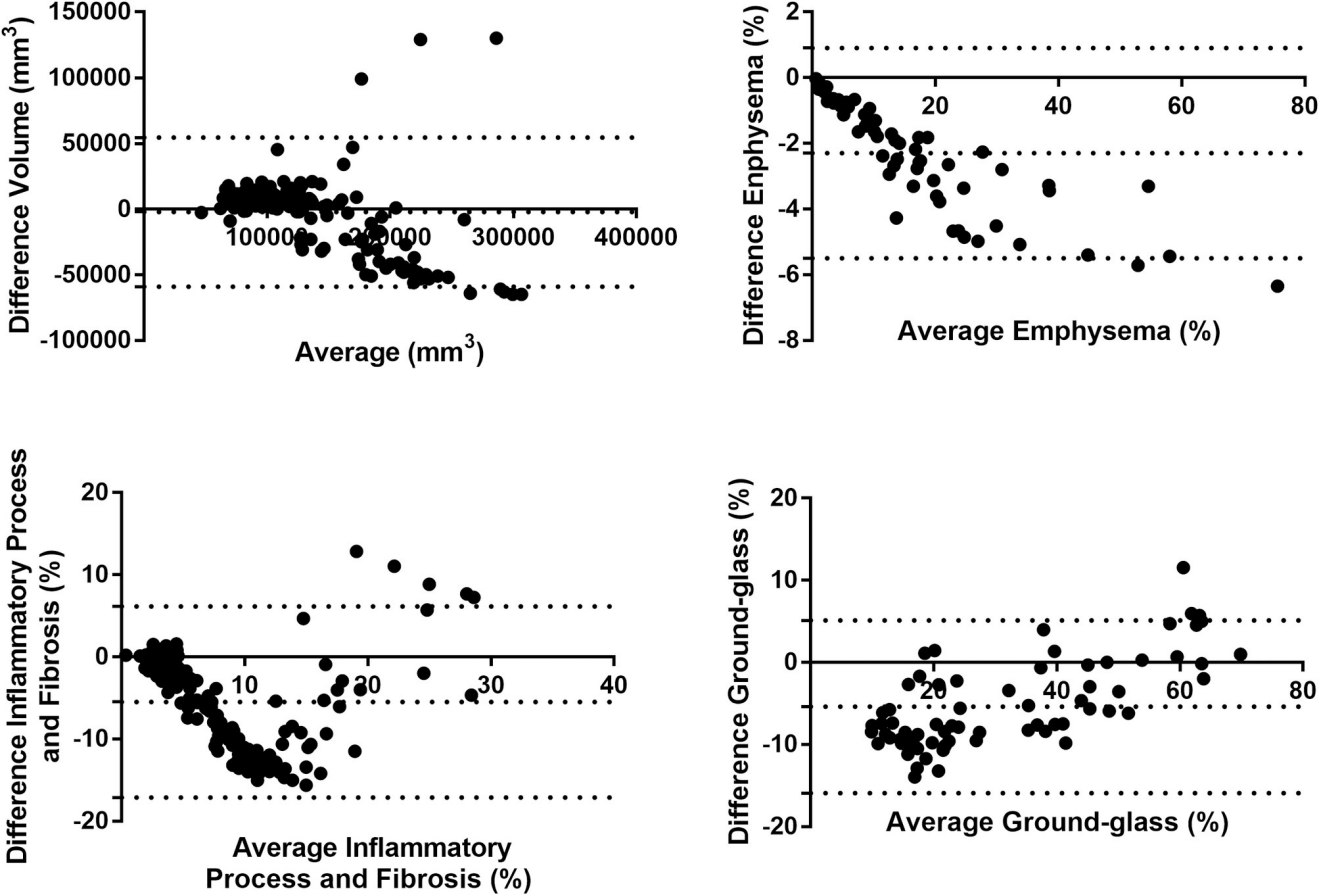
Bland–Altman plots comparing the difference the semi-automatic and the automatic algorithms. A—Lung volume, B—Emphysema, C—Inflammatory process and fibrosis, and D—Ground-glass opacities. Short dashed lines indicate the interval of 2 standard deviations, indicating an excellent level of statistical agreement between the results.

**Fig 5:**
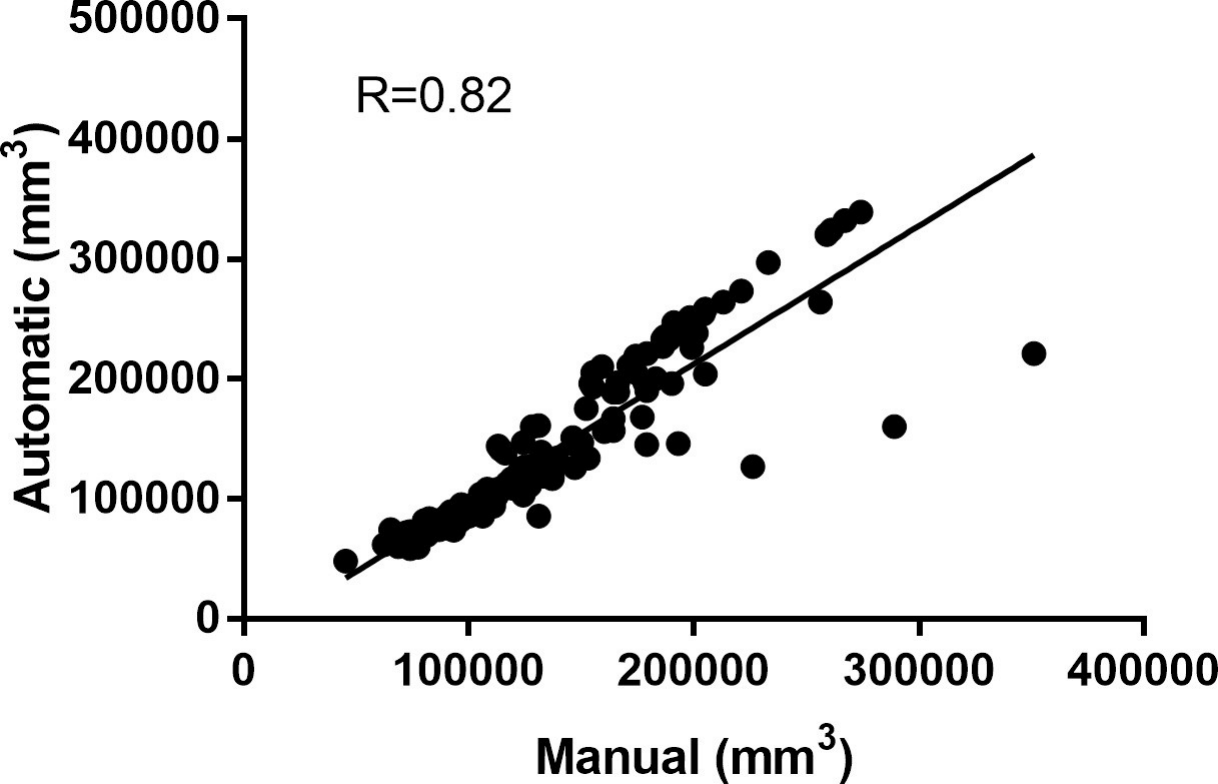
Linear regression comparing the automatic and semi-automatic algorithm regarding total lung volume among all patients.

In Fig 4 the Bland–Altman plots demonstrate the score of difference between the semi-automatic and automatic evaluations. The limits of agreement between automatic and semi-automatic algorithms for all patients involved in the study are shown in Fig 4A–4D. In this Figure, we compare the total pulmonary volume (Fig 4A), and the percentage of pulmonary volume compromised by emphysema (Fig 4B), inflammatory process and fibrosis (Fig 4C) and ground-glass opacities (Fig 4D). The relationship between the total volumes of all patients quantified is demonstrated in Fig 5 as a linear regression adjustment with an R value of 0.81.

## Discussion

In this work, we developed an automatic algorithm to quantify lung volume and other lung impairments (emphysema, inflammatory process and fibrosis and ground-glass opacities). This algorithm can provide the total lung volume and the volume of each impairment with great precision compared to the semi-automatic algorithm [17]. This algorithm is completely automatic. That means it does not rely on operator interventions. In the semi-automatic algorithm, the selection of the region of interest (ROI) was performed manually and required a radiologist with experience to define the pulmonary region. This process can take up to two hours to segment and then quantify a CT sequence with 60 slices. The automatic algorithm is capable of performing the same task in less than 1 minute with a standard performance personal computer as described in the methodology. However, it is possible to quantify a sequence with more than 300 slices in approximately 5 minutes. With a higher performance computer, this time can be reduced significantly. Those running times vary depending on the number of slices per patient.

The great difference between both algorithms occurs in the segmentation of the pulmonary area and volume, as the first is automatic and the second is manual in this step. This pulmonary delimitation directly influences the quantification process of the dilation and erosion operators that were applied in the algorithm, which contributes to different quantifications results of the impairments.

The algorithm makes no distinction between fibrosis and inflammatory process. The distinction between the two processes depends on the stage of the disease associated with clinical analyzes. In our results, they appear as a single impairment named inflammatory process and fibrosis, since they have similar Hounsfield Unit numbers.

In Fig 4A, the biases of Bland-Altman plots for the volume of all patients are considerably low (1.3% concerning the mean of the volumes) and the data has small dispersion. Fig 5 shows a good correlation between the methods (0.81) for volume estimation, with slope of 0.7. That indicates consistency between the results generated with the automatic and semi-automatic algorithm, the latter being validated previously [17], the difference being mainly attributed to a pixel-pixel count analysis for the automatic method, which generates a greater quantification than involving manual procedure. An accurate and automatic quantification of the pulmonary volume is important because this parameter is reflected in several clinical analyzes [12].

Fig 4B–4D shows the comparisons between methods of quantifying pulmonary involvement. Low dispersion for quantified impairments (inflammatory process and fibrosis, emphysema, and ground-glass) is observed. In addition, the biases are small (-2.3% for emphysema, -5.24% for inflammatory process / fibrosis and -5.4% for ground glass). These results represent the agreement between the automatic method and the already validated (semi-automatic) method for quantifying pulmonary structures. The differences found are mainly associated with the operator’s selection of regions (segmentation). Despite their experience, human operators select fewer points within their ROI to segment the lung region when compared to the automatic process performed through thresholds. That difference in each lung area may reflect in a larger difference when accounting for the entire sequence. We consider the automatic process more accurate as it does not depend on the subjective evaluation of the operator and evaluates the quantification process pixel by pixel.

With our study, it was possible to determine the percentage of impairments associated with different lung diseases. According to subjective reports [2], ground-glass opacities were the impairment found with the highest percentage in patients with SARS-CoV-2 (44%—Table 1). The quantification of ground-glass can support not only diagnosis but also clinical procedures to be conducted on the patient. These patients presented about 65% of pulmonary impairment (emphysema, inflammatory process and ground glass).

PCM causes pulmonary sequelae, destroying the lung parenchyma, which can be replaced by areas of fibrosis, emphysema, or a combination of both [21]. Our results show that in this group the lung is involved in about 39% of all cases (Table 1), confirm findings reported by [17] in addition to emphysema and fibrosis. Also, in this study, it was possible to quantify ground-glass opacities (22%) in PCM patients, quite high values. These values demonstrate the importance of their quantification and potentially require more attention in future clinical investigations.

The recognition of CT patterns described in pulmonary PCM could aid in the early diagnosis of PCM and the institution of a specific treatment [12]. CT scans may reveal alterations that are suggestive of the mycosis, while the disease is in an early stage. The most frequent CT findings consistent with the diagnosis include interlobular septal thickening, ground-glass opacities, a focal round area of ground-glass attenuation surrounded by a crescent or ring of consolidation (reversed halo sign) and irregular air–space enlargement [22].

In Marchiori et al. [23] series, the ground-glass pattern corresponded histologically to inflammatory infiltrate in alveolar septa, or, less commonly, to alveolar septal fibrosis.

The presence of fibrosis was characterized in this series by the findings of architectural distortion, bronchiectasis, and honeycombing, besides alveolar and interlobular septal thickening. The extent of the fibrotic phenomena is determined by the changes caused by the introduction of specific therapy and by the typical slow, chronic progression of the disease.

Chronic obstructive pulmonary disease (COPD) causes an inflammatory process producing changes in the lung parenchyma (pulmonary emphysema). Our result shows a great occurrence of emphysema (49% of the lung volume), which enables quantitative characterization of this disease. We emphasize that the measurement of lung volume is an important parameter to support other studies in COPD [24].

Although our exclusion criteria for no lung disease patients were strict, this group presented a reasonable percentage of emphysema (14%), which is related to the average age of the group and possible inherent and morbidities not included in the criteria for exclusion, demonstrating a good performance of the method to evaluate other classes of patients.

Therefore, the objective of the algorithm was to quantify lung involvement objectively and quickly, bringing information of affected areas and volumes with greater precision, also saving time of the radiologists. In future studies it could be used to follow disease progression in some patients as a prospective evaluation.

## Conclusion

The great contribution of this work was to demonstrate a tool capable of performing automatic quantifications of total lung volume and lung impairments. The algorithm is capable of quantifying the total lung volume as well as other lung impairments such as inflammatory process and fibrosis, emphysema, and ground-glass opacities in four groups of patients: no lung disease, SAR-COV-2, COPD and PCM. This automatic algorithm has an excellent performance of running time, and an great level of agreement when compared to a previously validated semi-automatic algorithm. We applied our algorithm in different diseases and with a high number of patients demonstrating that it is possible to quantify lung volume and impairments with low dispersion and high correlation in relation to the previous semi-automatic algorithm. Our approach provides a reliable quantification process for physicians, thus, impairments measurements contributes to support prognostic decisions. Our tool could be used in prospective serial studies, contributing to the patient’s clinical evaluation and treatment conduct.
